# The functional role of microRNAs in alcoholic liver injury

**DOI:** 10.1111/jcmm.12223

**Published:** 2014-01-08

**Authors:** Kelly McDaniel, Leonardo Herrera, Tianhao Zhou, Heather Francis, Yuyan Han, Phillip Levine, Emily Lin, Shannon Glaser, Gianfranco Alpini, Fanyin Meng

**Affiliations:** a Research, Central Texas Veterans Health Care SystemTemple, TX, USA; b Department of Medicine, Scott & White Digestive Disease Research Center, Texas A&M University Health Science Center and Scott & White HealthcareTemple, TX, USA; c Academic Operations, Scott & White HospitalTemple, TX, USA; d Texas Bioscience InstituteTemple, TX, USA

**Keywords:** alcoholic liver diseases, microRNAs, Kupffer cells, TLR4, TNF-α, LPS, apoptosis

## Abstract

The function of microRNAs (miRNAs) during alcoholic liver disease (ALD) has recently become of great interest in biological research. Studies have shown that ALD associated miRNAs play a crucial role in the regulation of liver-inflammatory agents such as tumour necrosis factor-alpha (TNF-α), one of the key inflammatory agents responsible for liver fibrosis (liver scarring) and the critical contributor of alcoholic liver disease. Lipopolysaccharide (LPS), a component of the cell wall of gram-negative bacteria, is responsible for TNF-α release by Kupffer cells. miRNAs are the critical mediators of LPS signalling in Kupffer cells, hepatocytes and hepatic stellate cells. Certain miRNAs, in particular miR-155 and miR-21, show a positive correlation in up-regulation of LPS signalling when they are exposed to ethanol. ALD is related to enhanced gut permeability that allows the levels of LPS to increase, leads to increased secretion of TNF-α by the Kupffer cells and subsequently promotes alcoholic liver injury through specific miRNAs. Meanwhile, two of the most frequently dysregulated miRNAs in steatohepatitis, miR-122 and miR-34a are the critical mediators in ethanol/LPS activated survival signalling during ALD. In this review, we summarize recent findings regarding the experimental and clinical aspects of functions of specific microRNAs, focusing mainly on inflammation and cell survival after ethanol/LPS treatment, and advances on the role of circulating miRNAs in human alcoholic disorders.

- Introduction
- LPS
- TLR4
- TNFα
- Kupffer cells and increase in TNFα secretion
- microRNAs
- miRNA and epigenetics
- miR-155 and ethanol exposure
- miR-155 and TNFα
- miR-155 and NF-κB
- miRNA and endotoxin altered permeability
- microRNA and hepatic cell survival
- Circulating miRNAs as stable blood-based markers for ALDs
- Conclusion

## Introduction

Alcoholic liver disease presents a global health concern 1. This disease ranges from alcoholic fatty liver and steatohepatitis to alcoholic cirrhosis, and includes hepatocellular carcinoma 2,3. It is the 12th leading cause of death in the United States, according to the National Institute on Alcohol Abuse and Alcoholism and accounted for a total of 14,364 deaths in 2007. Nearly 14 million Americans-abuse alcoholic or are alcoholics. Several million more adults could be on their way to alcohol problems. More notably, alcohol is implicated in more than 200 diseases and is a direct causal factor in 60 types of diseases and injury 4. The liver is the main site of alcohol metabolism and a major target organ of alcohol-induced injury 1. Alcoholic liver disease has been the leading cause of liver-related disease worldwide 5–7. Alcoholic liver disease is defined by scarring of the liver by the inflammatory agent tumour necrosis factor-alpha (TNF-α) which is secreted by Kupffer cells in the liver 8. The release of TNF-α is triggered by the binding of lipopolysaccharide (LPS) by toll-like receptor 4 (TLR4) on Kupffer cells 9. It is the translocation of these bacterial products in the lumen of the intestine that causes homeostatic imbalances in the liver. Because of the liver's importance to homeostasis and the worldwide prevalence of alcoholic liver disease, liver function has been given extra emphasis in biomedical research.

One control point regulating inflammatory response and cellular apoptosis in ALD involves microRNA targeting of critical inflammation/apoptosis signalling proteins. MicroRNAs are short functional RNAs that cause reduced expression of their target genes through post-transcriptional mechanisms. In general, this targeting involves imperfect base-pairing between the microRNA and the cognate mRNA target, resulting in altered protein production 10,11. In human ALDs, microRNAs are intimately involved in development and progression of liver injury, and act to alter expression of disease-related targets 12,13. Much attention has been devoted to microRNA function in recent years, in part because microRNA biology is still being elucidated, and in part because of their promise in diagnosis and treatment of disease. Modulation of microRNA function is an attractive emerging approach to ALD treatment, and studies to understand the underlying mechanisms of altered microRNA expression and functions are necessary to pursue this treatment strategy.

## LPS

Lipopolysaccharide exposure is initiated by the liver's detoxification process upon exposure to gram-negative bacterial cell wall 14–16. Lipopolysaccharide is released into the blood stream when gram-negative bacterial cells die or lyse and are then transported to the liver for detoxification 17. The LPS alone has been found to increase with alcoholic liver consumption when gut permeability is severely compromised. The experiments with alcohol-fed mice revealed that endotoxin levels increased in alcohol-fed mice when compared with normal mice 14–16. It was concluded from this experiment that such results were a direct result of the weakening of the intestinal tight junctions by ethanol 18. The excess of endotoxins is absorbed by the intestinal lumen and later passes through the liver, where monocytes and macrophages, such as Kupffer cells are then exposed to the toxin 19,20. In addition to the increased permeability of the intestinal lumen due to ethanol exposure, ethanol exposure also seems to be correlated with an increased amount of gram-negative bacterial growth in the lumen of alcoholics' intestines. Although this increase in bacterial growth is not certain, suggestive evidence has led to further research on the subject 21. Since alcohol can significantly increase the translocation of LPS from the gut. The study of these interactions may provide potential new targets for therapeutic intervention.

## TLR4

Toll-like receptors were first discovered in Drosophila and later as corresponding human homologs 9. One particular toll-like receptor, TLR4, identifies and binds LPS through its co-receptors CD14 or MD-2 22,23. MD-2 is a soluble protein that non-covalently associates with TLR4 and binds LPS directly to form a complex with LPS in the absence of TLRs 24. MD-2 has been shown to have increased concentrations in alcohol-fed mice 25. It is known to exist in a soluble form which, when in high concentrations, has shown to be an inhibitor of endotoxin-activated TNF-α secretion 9. CD14, however, is found in monocytes, macrophages, parenchymal cells and fibroblasts 26,27. Its use in the LPS induced TNF-α pathway was confirmed during CD14 inhibition in mice. CD14 inhibited mice became resistant to endotoxin shock 28. Upon contact with TLR4 receptors and co-receptors, LPS induces Kupffer cells to release TNF-α 6. Because of this, the TLR4 and LPS relation plays a key role in the mediation of inflammatory agents in the liver.

There is ample evidence for increased inflammatory cascade activation in ALD. Toll-like receptor 4 is expressed in all cell types of the liver; thus, gut-derived endotoxin can modulate the function of all liver cells in ALD 9,29,30. Over the years, compelling evidence has revealed that the TLR4-LPS signalling pathway plays a critical role in alcohol-induced liver injury 31. Both chronic and acute (or binge) alcohol use affects the various components of TLR4 signalling. There is increased expression of TLR4 and its co-receptors, as well as other TLRs, in ALD in mice 9. The studies in TLR4 mutant mice demonstrated protection from early ALD, and recent reports using TLR4 deficient mice validated the important role of TLR4 in the pathogenesis of ALD 25.

Some feedback mechanisms exist to limit LPS mediated toxic effects. Several soluble decoy receptors, such as soluble TLR4, and splice variants of signal-transduction proteins, including MyD88-s, IRAK-M and TAG, are the key regulators 32. Toll-like receptor 4 mediates LPS signalling with the assistance of its co-receptors, CD14 or MD-2 22,23. Lipopolysaccharide/TLR4 enlists the adaptor molecules MyD88 and TRIF, and subsequently activates downstream signalling pathways, respectively. Activation of NF-kB by TLR4-MyD88 complex enhances the production of pro-inflammatory cytokines, such as TNF-α, interleukin (IL)-6 and IL-1b 23. Tank Binding Kinase-1/IkB kinase-e/IKKi (TBK/IKKe) phosphorylation and activation of the interferon regulatory factor-3 by TRIF signalling lead to the production of IFN-γ-interferons 33. Within 4 days of ethanol exposure, there was a striking spike in expression of IFN-γ, along with TNF-α and IL-6 – prior to hepatic triglyceride accumulation or increased plasma alanine aminotransferase (ALT) activities, as well as before the induction of cytochrome P450 2E1 or oxidative stress 34. Therefore, activation of both of the pro-inflammatory and IFN-γ pathways could be either LPS/TLR4 dependent or independent, and evaluation of these specific pathways may have translational impact on ALD 9,34. microRNAs have also been introduced, such as miR-146a and miR-21, which is induced by LPS and negatively targets signalling proteins such as IRAK1, TRAF6 and PDCD4 at the post-transcriptional level 32,35,36.

## TNF-α

Tumour necrosis factor-alpha is an inflammatory agent in the liver which has been found to be a key contributor to alcoholic liver disease 5. Tumour necrosis factor-alpha is released by macrophages in the liver upon introduction of LPS, which binds to TLR4. The release of TNF-α causes inflammation, which leads to liver fibrosis 37. A recent study determined that the production of TNF-α in the liver by Kupffer cells was increased both by the introduction of ethanol and LPS individually. In this study, the levels of TNF-α increased when a dosage of LPS was introduced to the liver macrophages while keeping the ethanol levels unchanged. The reverse experiment was conducted where ethanol was increased and no additional LPS was introduced into the system. In both experiments the TNF-α production rose. Since LPS is the known component to activate the TLR4 pathway, this experiment confirmed that it is not ethanol's direct involvement in the pathway's activation, but rather ethanol's mediation of LPS and other factors that leads to the liver scarring 31. The TLR4 pathway plays a key role in the activation of TNF-α, but it is the TNF-α protein, which regulates the inflammation.

## Kupffer cells and increase in TNF-α secretion

Kupffer cells are resident macrophages in the liver that become activated upon recognition of an LPS signal both *in vivo* and *in vitro* 9,31. Kupffer cells were identified as a key component of alcoholic liver disease through studies of their activation and deactivation. *Inactivation of Kupffer cells* with gadolinium *chloride or clodronate* injection can ameliorate *alcohol-* induced liver disease 38,39. It was concluded, thereafter, that Kupffer cells are, in part, responsible for alcoholic liver disease 38,39. LPS signalling to TLR4 and its co-receptors stimulates Kupffer cells to release TNF-α, which causes scarring of liver tissue and ultimately alcoholic liver disease 31. The damage caused by TNF-α from Kupffer cells in the liver ranges from steatosis and inflammation to hepatocyte damage in alcoholic liver disease 40,41. There exist three particular miRNAs that regulate the production of TNF-α in Kupffer cells: miR-155 and miR-146a 36,42, although only one shows positive up-regulation of TNF-α: miR-155 43. As the major macrophage population of the body that has direct contact with the blood, Kupffer cells have been of great interest in recent research.

## microRNAs

**Fig 1:**
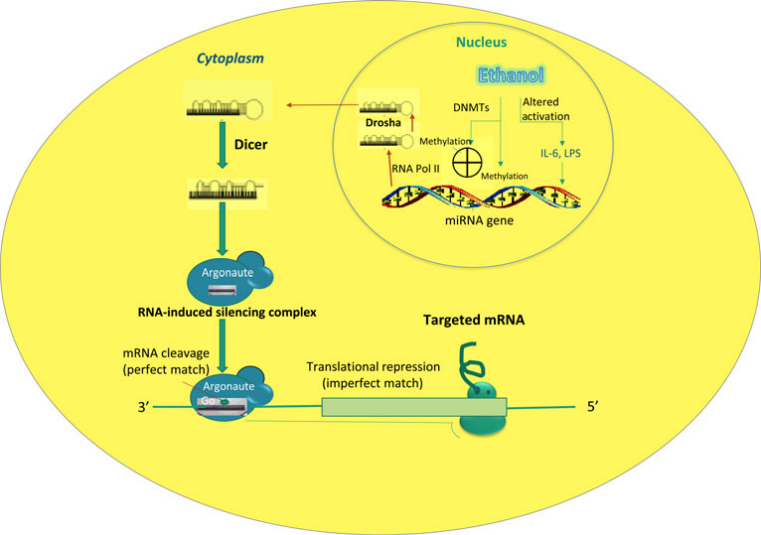
Aberrant expression and functional changes of specific miRNAs during alcoholic liver injury. During alcoholic liver disease, Ethanol affects miRNA expression by altering activation of transcription factors including lipopolysaccharide, interleukin-6 and tumour necrosis factor-alpha, and/or epigenetic modification enzymes such as methyltransferases, such as DNA methyltransferases 1 (DNMT1), DNMT3A and DNMT3B. miRNA precursors are cleaved by RNases Drosha and Dicer while undergoing transport from the nucleus to the cytoplasm. Association of the mature miRNA with an Argonaut protein (Ago) directs the complex to complementary target sequences in specific messenger RNAs. If the target is perfectly complementary to the miRNA, Argonaute 2, a ribonucleoprotein associated with the miRNA, can mediate its cleavage. However, the imperfect match between miRNA and target may only result in translational repression without mRNA alterations 104–106.

microRNAs are segments of RNA, which serve as epigenetic regulators. The first discovery and observation of miRNA's function occurred in 1993, and over the subsequent 10 years, 300+ miRNA sequences have been discovered. Today, ∼17,000 miRNAs have been described in 142 species. Of these, around 1000 are present in the human genome 44,45. MicroRNAs (miRNAs) belong to a group of non-protein-coding RNAs (ncRNAs) about 20–22 nucleotides long 46–48 that have been found to be key regulators of gene expression 5,6. They are transcribed from RNA polymerase II or Poly II. They begin their existence in the nucleus, and then travel to the cytoplasm where they mature (Fig. 1). They are responsible for the alternation of hundreds of genes by binding to the untranslated regions of mRNA 49. miRNAs have also been found to have influence on the modulation of methylation 50,51. Certain miRNAs have been linked to specific diseases such as hepatocellular carcinoma and various types of hepatitis. These miRNAs are the first responders when cells signal damage or pathogenic infection. They are important in the body's immune response. 6 Due to their vast amount of influence on the body, the study of miRNAs has gained a significant amount of interest in recent research.

## miRNA and epigenetics

Epigenetics, in its essence, defines the alternation of gene expression without disruption of DNA sequences. Epigenetic regulation is altered and manipulated by several factors, including DNA methylation, modifications of histones and RNA silencing by non-protein-coding RNAs (ncRNAs) 46,47. DNA methylation is an epigenetic modification that can regulate gene expression and is tightly regulated by at least three DNA methyltransferases (DNMT-1, DNMT-3A and DNMT-3B). Aberrant DNA methylation has been implicated in many human diseases including alcoholic liver disease 52–54. Alcohol consumption causes cellular injury. Recent developments indicate that ethanol induces epigenetic alterations, particularly acetylation, methylation of histones, and hypo-and hypermethylation of DNA 55–57. This has opened up a new area of interest in ethanol research and is providing novel insight into actions of ethanol at the nucleosome level in relation to gene expression and pathophysiological consequences. Although DNA methylation has been tightly linked to liver injury and poor disease outcome in many hepatic disorders, including human ALD, its application to ethanol-dependent ncRNA expression is novel. A better understanding of how ethanol interacts with specific DNA methyl transferases and contributes to aberrant ncRNA expression will clearly advance the field and increase our understanding of the mechanisms involved in the development of ALD.

Altered DNA methylation occurs after alcohol consumption during initial periods of alcohol abuse. Global hypomethylation of DNA in liver after long-term ethanol exposure has been reported 58. Regional hypomethylation of the c-myc gene occurs in the liver after long-term consumption of alcohol. Decreased DNA methylation with a concomitant decrease in DNMT activity after ethanol exposure of pregnant rats has been reported in foetal tissues 59. Decreased activity of DNMT has also been discovered in peripheral blood cells from ALDs. Moreover, ethanol consumption has also been shown to be associated with reduced DNMT transcript levels and altered methylation of imprinted DNA regions in sperm 60–62. In addition, chronic ethanol consumption can impair 1-carbon metabolism, consequently diminishing the availability of S-adenosyl-methionine. This methyl donor is required for both DNA and histone methylation 63,64. Ethanol-mediated reductions in DNA methylation could be expected to increase the expression of affected genes, including ncRNAs. Therefore, the intriguing possibility worthy of investigation is that epigenetic changes as a result of ethanol may account for altered expression of some miRNAs.

miRNA is also a modulator of epigenetics in the liver at a post-transcriptional level. It is responsible for gene expression regulation. They are responsible for the hindering of several translational elements to include: initiation, elongation, degradation, and degradation of target mRNA 65–67. The role of miRNA epigenesis does not only involve the regulation of LPS signalling or TNF-α production, miRNA is also a major component in regulating intestinal permeability 67. Ethanol has a prolonged effect on the regulation of miRNA (Table 1). Several studies showed that ethanol alters miRNA concentration in alcohol-fed mice. Several different miRNAs were found to be aberrantly expressed with ethanol exposure. While the predisposing risk factors and aetiologies of ALDs were varied, the deregulation of some specific miRNAs was commonly identified in the published studies, suggesting their importance in alcoholic liver injury. Among these, the over-expression of miR-21, miR-34a, miR-155, miR-320 and the under-expression of miR-122, miR-181a, miR-199a, miR-200a were reported by more than one publication. These miRNAs are described in Table 2. Three of the more notable ones are miR-122, miR-34a and miR-21. It accounts for over 70% of the liver's total miRNA content. The other notable one is, of course, miR-155, which regulates inflammatory agent secretion in the liver 68. One of the miRNA's responsible for maintaining intestinal permeability is the miR-122, which as was mentioned before is negatively affected by ethanol exposure. Ethanol also up-regulates miR-155, which is responsible for the mediation of LPS induced TNF-α secretion 69.

**Table 1:** miRNA gene expression studies in ALD.

Year	Profiling method	Main conclusions of the studies	References
2008	Microarray/Northern blot	HCC cases associated with alcohol consumption displayed a decrease in miR-126 expression	98
2009	qPCR	Ethanol-induced miR-199 down-regulation may contribute to augmented HIF-1alpha and ET-1 expression	99
2009	Microarray	Hepatic specimens from mice fed with an ethanol-containing diet (Lieber–DeCarli) indicated features of alcoholic steatohepatitis and had an increased expression of miR-320, miR-486, miR-705, and miR-1224 and a decreased expression for miR-27b, miR-214, miR-199a-3p, miR-182, miR-183, miR-200a, and miR-322	100
2009	qPCR	Expression of miR-375 was shown to be highly expressed and was shown to increase with alcohol consumption, suggesting that this miRNA could represent a molecular fingerprint of alcohol consumption	101
2011	qPCR	Chronic alcohol consumption increases miR-155 in macrophages *via* NF-κB and the increased miR-155 contribute to alcohol-induced elevation in TNF-α production *via* increased mRNA stability	31
2012	Microarray/Northern blot/qPCR	Methylation-associated miRNA, miR-34a, was increased in ethanol feeding mice liver	52
2012	qPCR	miR-217 is increased after ethanol treatment and is a specific target of ethanol action in the liver	91
2012	qPCR	The increase of miR-21 expression during liver regeneration is more robust in ethanol-fed rats	102
2013	Microarray/qPCR	Several miRNAs that were significantly altered by chronic EtOH feeding, including miR-34a, miR-103, miR-107 and miR-122 have been reported to play a role in regulating hepatic metabolism and the onset of these miRNA changes occurred gradually during the time course of EtOH feeding	103

**Table 2:** Most commonly dysregulated miRNAs in ALD.

miRNA	Chromosome location	Dysregulation	References
miR-122	18q21.3	Decreased/Increased	52,103
miR-125b	11q24.1	Decreased	103
miR-126	9q34.3	Decreased	98
miR-155	21q21.3	Increased	31,99
miR-181a	1q32.1	Decreased	52,100
miR-199a	1q24.3	Decreased	99,100
miR-200a	1p36.33	Decreased	100,103
miR-21	17q23.2	Increased	52,102
miR-217	2p16.1	Increased	91
miR-320	8p21.3	Increased	100,103
miR-34a	1p36.22	Increased	52,103
miR-375	2q35	Increased	101
miR-486	8p11.21	Increased	100
let-7b	22q13	Decreased	52

## miR-155 and ethanol exposure

**Fig 2:**
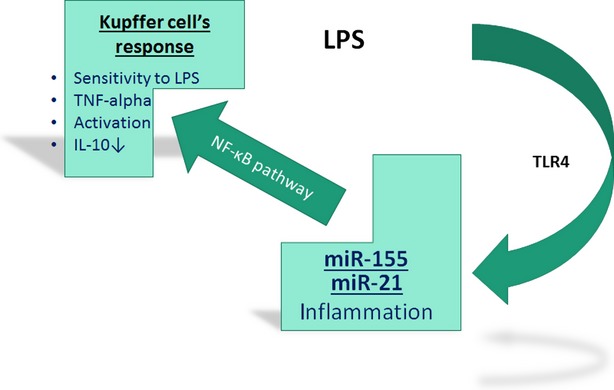
microRNAs mediated lipopolysaccharide (LPS)/toll-like receptor 4 signalling in Kupffer's cells during alcoholic liver injury. Alcohol consumption may increase gut permeability and subsequent bacterial or microbial translocation into intestinal lumen and result in the increase of LPS in the portal circulation. The excess of LPS in the liver affects Kupffer's cells through miR-155/miR-21, and in response there is the activation of NF-κB signaling as well as the alterations of its downstream effects.

miRNA is a known regulator of Kupffer cell response to LPS. Three miRNAs in particular, miR-155, miR-125b and miR-146a, have been of interest in studies and shown to be key contributors to LPS regulation 69. Only miR-155 and miR-146a, however, seemed to show up-regulation in macrophages such as Kupffer cells. Although both miR-146a and miR-155 was shown unregulated, only miR-155 showed to have a positive regulatory effect on the secretion of the inflammatory agent TNF-α by enhancing its translation (Fig. 2). Because of these findings, miR-155 received particular attention in the study of miRNAs' effect on alcoholic liver disease 42,70. Recent studies have shown that alcohol targets miR-155 and induces miR-155 in RAW264.7 macrophages and Kupffer cells. It was concluded from this study that miR-155 is directly correlated with TNF-α levels. Alcohol-fed mice showed increased levels of TNF-α and miR-155 31. This same study tested other miRNA's such as miR-125b and miR146a, but only miR-155 showed a significant affect in the inflammatory response of RAW 264.7 macrophages 31. Similar studies have also shown that alcohol treatment increases miR-155 in the same RAW 264.7 macrophages *in vitro*. Since miR-155 plays such a crucial role in the stimulation of LPS-induced TNF-α production, this study stated that the inhibition of miR-155 prevents alcohol-induced sensitization to LPS. Inversely, it was found that the up-regulation of miR-155 increased Kupffer cell sensitivity to LPS signalling. Experimentation on alcohol-fed mice showed that alcohol exposure up-regulates miR-155 43, which leads to Kupffer macrophage activation through miRNA mediation, causing the cells to release more TNF-α 31. Earlier reports using pharmacological inhibitors of NF-κB 31 have demonstrated that alcohol-induced up-regulation of miR-155 is *via* NF-κB, thus linking NF-κB with miR-155.

## miR-155 and TNF-α

The up-regulation of TNF-α production in Kupffer cells by LPS and alcohol was determined in two individual experiments: one in which LPS alone was increased, the other in which ethanol exposure alone was increased. In both experiments the production of TNF-α by resident macrophages was increased. A tertiary experiment also proved that miR-155 is the bridge step between ethanol and LPS up-regulation. In this experiment, miR-155 induced cells produced more TNF-α than the controls with no miR-155 overexposure. Lipopolysaccharide levels were also higher though no LPS was introduced. This led to the conclusion that miR-155 is directly involved in LPS-induced TNF-α production, not ethanol 31. The effects of LPS on Kupffer cells, however, become saturated past a point where alcohol-induced miR-155 no longer has any significant effect on the production on TNF-α after LPS stimulation 31.

## miR-155 and NF-κB

Studies have linked the inhibition of miR-155 to the NF-κB inhibitor. NF-κB inhibitors have proven to mediate the up-regulation of miR-155 in Kupffer cells 31. Furthermore, NF-κB has shown to be activated with chronic ethanol exposure and LPS stimulation 42,71. This leads to the studies to prove that miR-155 is regulated by NF-κB 72. NF-κB is a heterodimeric transcription factor usually composed of p50 and p65 subunits and is a pleiotropic regulator of various inflammatory and immune responses during alcoholic liver injury. Under unactivated condition, p50/p65 dimers are sequestered in the cytoplasm bound to its inhibitors, the IκBs, which prevent the translocation into the nucleus. Following various stimulations, the IκBs are rapidly degraded, activating NF-κB. The active form of NF-κB rapidly translocates into the nucleus, binding to consensus sequences in the promoter/enhancer region of various genes, promoting their transcription 73. The increase in NF-κB nuclear binding activity of p65/p50 and p50/p50 in prolonged alcohol treatment has been demonstrated, the same as the increase in LPS-induced NF-κB activation. The NF-κB and LPS activation relation was proven during an experiment where NF-κB was inhibited by MG-132 or Bay11-1782. The inhibition of NF-κB decreased miR-155 in ethanol, LPS and alcohol + LPS treated macrophages. This gave the conclusion that NF-κB is indeed the mediator of miR-155 expression in alcohol-induction 31.

## miRNA and endotoxin altered permeability

Another regulator of gut-endotoxin permeability, other than ethanol, is miRNA. It was discovered that miRNA increases the permeability of the intestinal lumen in a similar way to ethanol. miRNA, however, does so by affecting the Zonula occludens 1 (ZO-1) protein negatively to induce intestinal lumen permeability. The ZO-1 protein is a critical component that insures the permeable response of the intestinal lumen to endotoxins. Just like with alcohol, the permeability of the intestinal lumen leads to higher absorption of endotoxins that are later transported to the liver to be detoxified. In this process, the detoxification of these endotoxins leads to an LPS-TNF-α chain reaction that causes alcoholic liver disease. In addition to its effects on gut permeability, ethanol also up-regulates miRNA responsible for the reduction of lumen permeability 74. One particular miRNA, miR-212, has been identified as a contributor to the loss of tight junctions in the intestinal lumen through the ZO-1 protein. Another miRNA strand identifies as a key contributor to endotoxin permeability is the miR-122a, which also interacts with ZO-1 protein to regulate lumen permeability 6. Such findings suggest that miRNA has more than one role in the development of alcoholic liver disease, first as a mediator of the endotoxins that activate the inflammatory scarring agents and then as a mediator to such specific signalling in liver macrophages.

## microRNA and hepatic cell survival

Several investigations have demonstrated the regeneration and remodelling potentials of the adult cells in the liver after injury including hepatocytes and cholangiocytes and with newer studies showing the plasticity of hepatic stellate cell (HSC) and perhaps other cell types as well 75–78. The persistence of endotoxin during ALDs not only activates the liver immune cells of the liver, but also affects the function of other liver cells (hepatocytes, cholangiocytes and HSCs) 24,78. Habitual alcohol consumption promotes hepatocyte death and inhibits the proliferation of mature hepatocytes that survive, leading to chronic liver damage. Alcoholic liver damage is generally accompanied by a ductular reaction that is characterized by periportal accumulation of atypical cholangiocytes. As in many other types of chronic liver disease 79, in alcoholic liver disease the intensity of this ductular reaction closely parallels the severity of liver injury 80. Cholangiocytes and HSCs have been defined as unique subpopulations in ALDs that possess the ability to initiate regeneration processes as well as liver fibrosis 78. Although the evidence has been provided to support the role of liver parenchymal and HSC in ALDs, the identity and functions of bile duct cells remains a mystery. Changes in the survival and remodelling activities may be used to characterize certain liver regeneration and fibrotic processes. The new and innovative technique to functionally characterize the remodelling properties of specific hepatic cells may ultimately allow the development of new diagnostic and therapeutic strategies for ALD patients.

**Fig 3:**
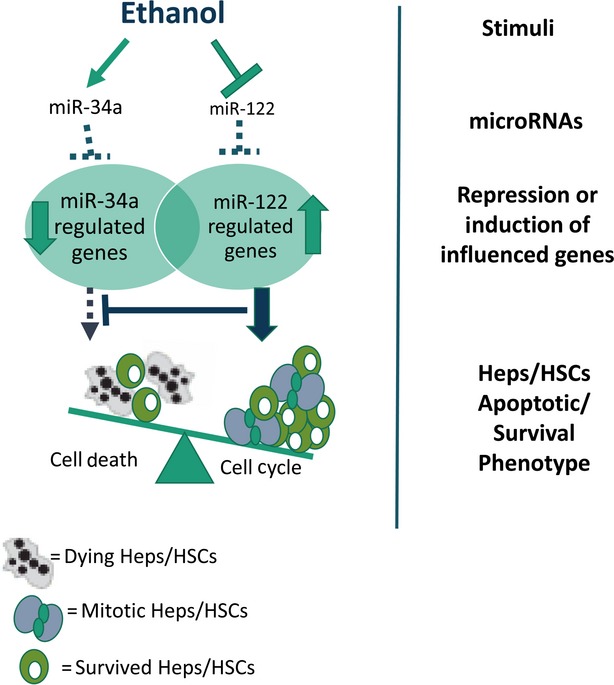
microRNA mediated survival mechanisms in alcoholic liver injury. miR-34a is anti-apoptotic, while miR-122, the liver specific miRNA, is the critical regulator of cell cycle. In normal liver, miR-34a and miR-122 cooperatively repress gene expression to balance cell survival and proliferation. Ethanol increases miR-34a and decreases miR-122 in the liver, resulting in altered target gene expressions, and consequently, increased cell proliferation while maintaining overall apoptosis resistance.

The key pathophysiological features of ALD are altered lipid metabolism and hepatocyte apoptosis. The results of recent studies show a significant down-regulation of miR-122, a liver-specific miRNA 81,82, which is important for normal lipid metabolism, and the marked up-regulation of miR-34a, a critical regulator of apoptosis 83, in the livers of mice fed with ethanol (Fig. 3). Several recent reports have shown that miRNAs miR-122 and miR-34a are two of the most frequently dysregulated miRNAs in steatohepatitis 84,85. Serum/plasma miR-122 has been correlated with ALT increases in the liver damage caused by alcohol, and was predominantly associated with the exosome-rich fraction 86. Both miR-122 and miR-34a were aberrantly expressed in both alcoholic steatohepatitis and non-alcoholic fatty liver disease 52,84,87. Sirtuin (silent mating type information regulation 2 homolog) 1 (SIRT1) is a verified target protein of miR-34a 88. SIRT1 plays an important role in protecting cells from cellular oxidative stress and DNA damage 89,90. Specific miRNA promotes ethanol-induced fat accumulation in hepatocytes by down-regulating SIRT1 91. Once SIRT1 is activated, SIRT1 deacetylates histones and histone methyl-transferases. SIRT1 also deacetylates a variety of non-histone target proteins, such as p53, the retinoblastoma protein (Rb), FoxO transcription factors, Ku70, NF-kB and PGC-1alpha. SIRT1-mediated deacetylation of Lys382 decreases p53-mediated transcriptional activation and reduced the downstream protein such as p21 and PUMA levels 92. Therefore, SIRT1 mediates the survival of cells during periods of severe stress through the inhibition of apoptosis. Overexpression of miR-34a decreased SIRT1 expression, leading to an increase in acetylated p53 levels and p53 targets, such as p21 and PUMA 93. miR-34a overexpression also induced apoptosis in cancer cells expressing p53, but not cancer cells not expressing p53. These results suggest that miR-34a induces apoptosis in part through a pathway that involves: miR-34a—SIRT1—p53 acetylation 93. However, the other relevant targets of miR-34a largely remain to be identified. Luciferase-based analysis has implicated E2F3, Foxp1, the Notch1 receptor, as well as its ligand Delta1, as potential miR-34a targets 94.

## Circulating miRNAs as stable blood-based markers for ALDs

The development of minimally invasive tests for the detection and monitoring of ALD could greatly reduce the worldwide health burden of alcoholic liver injury. The demonstration that miRNA profiles could reveal smoke-related effects in the liver of mice exposed to ethanol established a proof-of-principle for the use of miRNAs to evaluate early steps of ALD process, which could be extended to other risk factors. However, the most useful approach would be to distinguish specific biomarkers circulating in the bloodstream before alcoholic liver injury become clinically apparent. To this end, miRNAs can potentially become useful biomarkers for human ALD. Actually, deregulated miRNAs are found in liver as well as in the blood of ALD patients. Because of their innate stability, miRNAs may be detected blood based assays for ALD. miRNAs are present in plasma in a stable form, making them feasible biomarkers for the detection of ALD and other liver disorders 95,96. Recent proof-of-principle studies established that the analysis of miRNA expression in serum or plasma may be a promising approach for blood-based diagnosis of a number of human ALDs and other liver diseases 86,97. These studies suggest that the effect of ethanol consumption may be revealed by quantifying miRNAs circulating in serum/plasma. Further investigations will explore possibilities for circulating miRNAs as stable blood-based diagnostic markers and predicting progression for human ALDs.

## Conclusion

The role of miRNA in alcoholic liver disease is certainly a crucial one. In the past decade and in recent years its role in alcoholic liver disease has found keen interest. miRNA's interaction with alcohol plays a pivotal role in alcohol-induced liver scarring and fibrosis. The process that leads to alcoholic liver disease begins, of course, with ethanol consumption and exposure. Ethanol exposed mice have shown loss of permeability of the intestinal lumen. This permeability alternation occurs in two ways: First, with the direct influence of ethanol on the permeability of the intestinal lumen and allowing endotoxins to enter the liver. Second, ethanol induces miRNA, a known mediator of intestinal permeability, in the intestinal lumen to do the same. Once in the liver these endotoxins, produced by the decomposition of gram-negative bacteria and pathogens, follow a pathway that leads to liver inflammation. The endotoxins that are taken in by the intestinal lumen find their way to the liver, where they are detoxified. The detoxification of the endotoxins from gram-negative bacteria releases a particular kind of endotoxin called LPS, which once in the liver, attaches to TLR4, and sets in action the pathway that leads to alcoholic liver disease. Toll-like receptor 4 is not composed of two coreceptors, MD-2 and CD14, but these two proteins may be required for response of cell to LPS stimulation *via* TLR4. Both of these receptors have been identified as key components of alcoholic liver disease since both their deactivations help reduce progression of the disease. Upon activation of TLR4, macrophages in the liver release inflammatory agents that cause liver fibrosis. One particular macrophage of interest is the Kupffer cell, which releases TNF-α. The role miRNA takes part in this process involves the regulation of the LPS signalling to the TLR4 receptor. It is miRNA that regulates the LPS reception in Kupffer cells. It is also known that ethanol regulates miRNA (miR-122 and miR-34a) in the liver that contributes to hepatocytes/HSC survival. Therefore, as alcohol consumption increases, so does miRNA in the liver and thus the reception of LPS and TNF-α release. Although still under study, microRNAs have been shown to play a pivotal role in the development, causation and regulation of alcoholic liver disease.

## References

[b1] Massey VL, Arteel GE (2012). Acute alcohol-induced liver injury. Front Physiol.

[b2] Adachi M, Brenner DA (2005). Clinical syndromes of alcoholic liver disease. Dig Dis.

[b3] Tilg H, Day CP (2007). Management strategies in alcoholic liver disease. Nat Clin Pract Gastroenterol Hepatol.

[b4] Pompili M, Serafini G, Innamorati M (2010). Suicidal behavior and alcohol abuse. Int J Environ Res Public Health.

[b5] Miranda RC, Pietrzykowski AZ, Tang Y (2010). MicroRNAs: master regulators of ethanol abuse and toxicity?. Alcohol Clin Exp Res.

[b6] Bala S, Szabo G (2012). MicroRNA signature in alcoholic liver disease. Int J Hepatol.

[b7] Thompson KJ, McKillop IH, Schrum LW (2011). Targeting collagen expression in alcoholic liver disease. World J Gastroenterol.

[b8] Mello T, Polvani S, Galli A (2009). Peroxisome proliferator-activated receptor and retinoic x receptor in alcoholic liver disease. PPAR Res.

[b9] Szabo G, Bala S (2010). Alcoholic liver disease and the gut-liver axis. World J Gastroenterol.

[b10] Ambros V (2004). The functions of animal microRNAs. Nature.

[b11] Ambros V (2011). MicroRNAs and developmental timing. Curr Opin Genet Dev.

[b12] Gao B, Bataller R (2011). Alcoholic liver disease: pathogenesis and new therapeutic targets. Gastroenterology.

[b13] Szabo G, Petrasek J, Bala S (2012). Innate immunity and alcoholic liver disease. Dig Dis.

[b14] Keshavarzian A, Farhadi A, Forsyth CB (2009). Evidence that chronic alcohol exposure promotes intestinal oxidative stress, intestinal hyperpermeability and endotoxemia prior to development of alcoholic steatohepatitis in rats. J Hepatol.

[b15] Mathurin P, Deng QG, Keshavarzian A (2000). Exacerbation of alcoholic liver injury by enteral endotoxin in rats. Hepatology.

[b16] Bode C, Kugler V, Bode JC (1987). Endotoxemia in patients with alcoholic and non-alcoholic cirrhosis and in subjects with no evidence of chronic liver disease following acute alcohol excess. J Hepatol.

[b17] Hellman J, Loiselle PM, Tehan MM (2000). Outer membrane protein A, peptidoglycan-associated lipoprotein, and murein lipoprotein are released by Escherichia coli bacteria into serum. Infect Immun.

[b18] Rao RK (1998). Acetaldehyde-induced increase in paracellular permeability in Caco-2 cell monolayer. Alcohol Clin Exp Res.

[b19] Wheeler MD, Kono H, Yin M (2001). The role of Kupffer cell oxidant production in early ethanol-induced liver disease. Free Radic Biol Med.

[b20] Thakur V, McMullen MR, Pritchard MT (2007). Regulation of macrophage activation in alcoholic liver disease. J Gastroenterol Hepatol.

[b21] Bode C, Bode JC (2003). Effect of alcohol consumption on the gut. Best Pract Res Clin Gastroenterol.

[b22] Takeda K, Akira S (2004). TLR signaling pathways. Semin Immunol.

[b23] da Silva Correia J, Soldau K, Christen U (2001). Lipopolysaccharide is in close proximity to each of the proteins in its membrane receptor complex. transfer from CD14 to TLR4 and MD-2. J Biol Chem.

[b24] Mandrekar P, Szabo G (2009). Signalling pathways in alcohol-induced liver inflammation. J Hepatol.

[b25] Hritz I, Mandrekar P, Velayudham A (2008). The critical role of toll-like receptor (TLR) 4 in alcoholic liver disease is independent of the common TLR adapter MyD88. Hepatology.

[b26] Antal-Szalmas P, Strijp JA, Weersink AJ (1997). Quantitation of surface CD14 on human monocytes and neutrophils. J Leukoc Biol.

[b27] Liu S, Khemlani LS, Shapiro RA (1998). Expression of CD14 by hepatocytes: upregulation by cytokines during endotoxemia. Infect Immun.

[b28] Haziot A, Ferrero E, Kontgen F (1996). Resistance to endotoxin shock and reduced dissemination of gram-negative bacteria in CD14-deficient mice. Immunity.

[b29] Nath B, Szabo G (2009). Alcohol-induced modulation of signaling pathways in liver parenchymal and nonparenchymal cells: implications for immunity. Semin Liver Dis.

[b30] Guo J, Friedman SL (2010). Toll-like receptor 4 signaling in liver injury and hepatic fibrogenesis. Fibrogenesis Tissue Repair.

[b31] Bala S, Marcos M, Kodys K (2011). Upregulation of microRNA-155 in macrophages contributes to increased tumor necrosis factor alpha (TNF{alpha}) production *via* increased mRNA half-life in alcoholic liver disease. J Biol Chem.

[b32] Sheedy FJ, Palsson-McDermott E, Hennessy EJ (2010). Negative regulation of TLR4 *via* targeting of the proinflammatory tumor suppressor PDCD4 by the microRNA miR-21. Nat Immunol.

[b33] Kawai T, Takeuchi O, Fujita T (2001). Lipopolysaccharide stimulates the MyD88-independent pathway and results in activation of IFN-regulatory factor 3 and the expression of a subset of lipopolysaccharide-inducible genes. J Immunol.

[b34] Roychowdhury S, McMullen MR, Pritchard MT (2009). An early complement-dependent and TLR-4-independent phase in the pathogenesis of ethanol-induced liver injury in mice. Hepatology.

[b35] Nahid MA, Pauley KM, Satoh M (2009). miR-146a is critical for endotoxin-induced tolerance: IMPLICATION IN INNATE IMMUNITY. J Biol Chem.

[b36] Taganov KD, Boldin MP, Chang KJ (2006). NF-kappaB-dependent induction of microRNA miR-146, an inhibitor targeted to signaling proteins of innate immune responses. Proc Natl Acad Sci USA.

[b37] Hill DB, Barve S, Joshi-Barve S (2000). Increased monocyte nuclear factor-kappaB activation and tumor necrosis factor production in alcoholic hepatitis. J Lab Clin Med.

[b38] Koop DR, Klopfenstein B, Iimuro Y (1997). Gadolinium chloride blocks alcohol-dependent liver toxicity in rats treated chronically with intragastric alcohol despite the induction of CYP2E1. Mol Pharmacol.

[b39] Thurman RG (1998). II. Alcoholic liver injury involves activation of Kupffer cells by endotoxin. Am J Physiol Gastrointest Liver Physiol.

[b40] Iimuro Y, Gallucci RM, Luster MI (1997). Antibodies to tumor necrosis factor alfa attenuate hepatic necrosis and inflammation caused by chronic exposure to ethanol in the rat. Hepatology.

[b41] Uesugi T, Froh M, Arteel GE (2002). Role of lipopolysaccharide-binding protein in early alcohol-induced liver injury in mice. J Immunol.

[b42] Tili E, Michaille JJ, Cimino A (2007). Modulation of miR-155 and miR-125b levels following lipopolysaccharide/TNF-alpha stimulation and their possible roles in regulating the response to endotoxin shock. J Immunol.

[b43] Szabo G, Bala S, Petrasek J (2010). Gut-liver axis and sensing microbes. Dig Dis.

[b44] Lee RC, Feinbaum RL, Ambros V (1993). The C. elegans heterochronic gene lin-4 encodes small RNAs with antisense complementarity to lin-14. Cell.

[b45] Griffiths-Jones S (2004). The microRNA registry. Nucleic Acids Res.

[b46] Moss TJ, Wallrath LL (2007). Connections between epigenetic gene silencing and human disease. Mutat Res.

[b47] Rodenhiser D, Mann M (2006). Epigenetics and human disease: translating basic biology into clinical applications. CMAJ.

[b48] Bejarano F, Bortolamiol-Becet D, Dai Q (2012). A genome-wide transgenic resource for conditional expression of Drosophila microRNAs. Development.

[b49] Bartel DP (2009). MicroRNAs: target recognition and regulatory functions. Cell.

[b50] Sinkkonen L, Hugenschmidt T, Berninger P (2008). MicroRNAs control *de novo* DNA methylation through regulation of transcriptional repressors in mouse embryonic stem cells. Nat Struct Mol Biol.

[b51] Garzon R, Liu S, Fabbri M (2009). MicroRNA-29b induces global DNA hypomethylation and tumor suppressor gene reexpression in acute myeloid leukemia by targeting directly DNMT3A and 3B and indirectly DNMT1. Blood.

[b52] Meng F, Glaser S, Francis H (2012). Epigenetic regulation of miR-34a expression in alcoholic liver injury. Am J Pathol.

[b53] Kutay H, Klepper C, Wang B (2012). Reduced susceptibility of DNA methyltransferase 1 hypomorphic (Dnmt1N/+) mice to hepatic steatosis upon feeding liquid alcohol diet. PLoS ONE.

[b54] Davison JM, Mellott TJ, Kovacheva VP (2009). Gestational choline supply regulates methylation of histone H3, expression of histone methyltransferases G9a (Kmt1c) and Suv39h1 (Kmt1a), and DNA methylation of their genes in rat fetal liver and brain. J Biol Chem.

[b55] Pogribny IP, Tryndyak VP, Bagnyukova TV (2009). Hepatic epigenetic phenotype predetermines individual susceptibility to hepatic steatosis in mice fed a lipogenic methyl-deficient diet. J Hepatol.

[b56] Oliva J, Dedes J, Li J (2009). Epigenetics of proteasome inhibition in the liver of rats fed ethanol chronically. World J Gastroenterol.

[b57] Petrak J, Myslivcova D, Man P (2007). Proteomic analysis of hepatic iron overload in mice suggests dysregulation of urea cycle, impairment of fatty acid oxidation, and changes in the methylation cycle. Am J Physiol Gastrointest Liver Physiol.

[b58] Shukla SD, Aroor AR (2006). Epigenetic effects of ethanol on liver and gastrointestinal injury. World J Gastroenterol.

[b59] Bonsch D, Lenz B, Fiszer R (2006). Lowered DNA methyltransferase (DNMT-3b) mRNA expression is associated with genomic DNA hypermethylation in patients with chronic alcoholism. J Neural Transm.

[b60] Bielawski DM, Abel EL (2002). The effect of administering ethanol as single vs. divided doses on blood alcohol levels in the rat. Neurotoxicol Teratol.

[b61] Bielawski DM, Zaher FM, Svinarich DM (2002). Paternal alcohol exposure affects sperm cytosine methyltransferase messenger RNA levels. Alcohol Clin Exp Res.

[b62] Ouko LA, Shantikumar K, Knezovich J (2009). Effect of alcohol consumption on CpG methylation in the differentially methylated regions of H19 and IG-DMR in male gametes: implications for fetal alcohol spectrum disorders. Alcohol Clin Exp Res.

[b63] El-Moselhy MA, Abdel-Hamid NM, Abdel-Raheim SR (2009). Gastroprotective effect of nicorandil in indomethacin and alcohol-induced acute ulcers. Appl Biochem Biotechnol.

[b64] Shukla SD, Velazquez J, French SW (2008). Emerging role of epigenetics in the actions of alcohol. Alcohol Clin Exp Res.

[b65] Nottrott S, Simard MJ, Richter JD (2006). Human let-7a miRNA blocks protein production on actively translating polyribosomes. Nat Struct Mol Biol.

[b66] Humphreys DT, Westman BJ, Martin DI (2005). MicroRNAs control translation initiation by inhibiting eukaryotic initiation factor 4E/cap and poly(A) tail function. Proc Natl Acad Sci USA.

[b67] Liu J, Valencia-Sanchez MA, Hannon GJ (2005). MicroRNA-dependent localization of targeted mRNAs to mammalian P-bodies. Nat Cell Biol.

[b68] Whittaker R, Loy PA, Sisman E (2010). Identification of MicroRNAs that control lipid droplet formation and growth in hepatocytes *via* high-content screening. J Biomol Screen.

[b69] O'Connell RM, Taganov KD, Boldin MP (2007). MicroRNA-155 is induced during the macrophage inflammatory response. Proc Natl Acad Sci USA.

[b70] Perry MM, Moschos SA, Williams AE (2008). Rapid changes in microRNA-146a expression negatively regulate the IL-1beta-induced inflammatory response in human lung alveolar epithelial cells. J Immunol.

[b71] Mandrekar P, Bala S, Catalano D (2009). The opposite effects of acute and chronic alcohol on lipopolysaccharide-induced inflammation are linked to IRAK-M in human monocytes. J Immunol.

[b72] Rai D, Karanti S, Jung I (2008). Coordinated expression of microRNA-155 and predicted target genes in diffuse large B-cell lymphoma. Cancer Genet Cytogenet.

[b73] Fuseler JW, Merrill DM, Rogers JA (2006). Analysis and quantitation of NF-kappaB nuclear translocation in tumor necrosis factor alpha (TNF-alpha) activated vascular endothelial cells. Microsc Microanal.

[b74] Tang Y, Banan A, Forsyth CB (2008). Effect of alcohol on miR-212 expression in intestinal epithelial cells and its potential role in alcoholic liver disease. Alcohol Clin Exp Res.

[b75] Wang Y, Yao HL, Cui CB (2010). Paracrine signals from mesenchymal cell populations govern the expansion and differentiation of human hepatic stem cells to adult liver fates. Hepatology.

[b76] Scholten D, Osterreicher CH, Scholten A (2010). Genetic labeling does not detect epithelial-to-mesenchymal transition of cholangiocytes in liver fibrosis in mice. Gastroenterology.

[b77] Pintilie DG, Shupe TD, Oh SH (2010). Hepatic stellate cells' involvement in progenitor-mediated liver regeneration. Lab Invest.

[b78] Jung Y, Brown KD, Witek RP (2008). Accumulation of hedgehog-responsive progenitors parallels alcoholic liver disease severity in mice and humans. Gastroenterology.

[b79] Roskams T, Desmet V (1998). Ductular reaction and its diagnostic significance. Semin Diagn Pathol.

[b80] Roskams T, Yang SQ, Koteish A (2003). Oxidative stress and oval cell accumulation in mice and humans with alcoholic and nonalcoholic fatty liver disease. Am J Pathol.

[b81] Lynn FC (2009). Meta-regulation: microRNA regulation of glucose and lipid metabolism. Trends Endocrinol Metab.

[b82] Girard M, Jacquemin E, Munnich A (2008). miR-122, a paradigm for the role of microRNAs in the liver. J Hepatol.

[b83] Raver-Shapira N, Marciano E, Meiri E (2007). Transcriptional activation of miR-34a contributes to p53-mediated apoptosis. Mol Cell.

[b84] Cheung O, Puri P, Eicken C (2008). Nonalcoholic steatohepatitis is associated with altered hepatic MicroRNA expression. Hepatology.

[b85] Li S, Chen X, Zhang H (2009). Differential expression of microRNAs in mouse liver under aberrant energy metabolic status. J Lipid Res.

[b86] Bala S, Petrasek J, Mundkur S (2012). Circulating microRNAs in exosomes indicate hepatocyte injury and inflammation in alcoholic, drug-induced, and inflammatory liver diseases. Hepatology.

[b87] Min HK, Kapoor A, Fuchs M (2012). Increased hepatic synthesis and dysregulation of cholesterol metabolism is associated with the severity of nonalcoholic fatty liver disease. Cell Metab.

[b88] Lee J, Padhye A, Sharma A (2010). A pathway involving farnesoid X receptor and small heterodimer partner positively regulates hepatic sirtuin 1 levels *via* microRNA-34a inhibition. J Biol Chem.

[b89] He W, Wang Y, Zhang MZ (2010). Sirt1 activation protects the mouse renal medulla from oxidative injury. J Clin Invest.

[b90] Shen Z, Liang X, Rogers CQ (2010). Involvement of adiponectin-SIRT1-AMPK signaling in the protective action of rosiglitazone against alcoholic fatty liver in mice. Am J Physiol Gastrointest Liver Physiol.

[b91] Yin H, Hu M, Zhang R (2012). MicroRNA-217 promotes ethanol-induced fat accumulation in hepatocytes by down-regulating SIRT1. J Biol Chem.

[b92] Li Z, Chen L, Kabra N (2009). Inhibition of SUV39H1 methyltransferase activity by DBC1. J Biol Chem.

[b93] Yamakuchi M, Ferlito M, Lowenstein CJ (2008). miR-34a repression of SIRT1 regulates apoptosis. Proc Natl Acad Sci USA.

[b94] Lewis BP, Shih IH, Jones-Rhoades MW (2003). Prediction of mammalian microRNA targets. Cell.

[b95] Mitchell PS, Parkin RK, Kroh EM (2008). Circulating microRNAs as stable blood-based markers for cancer detection. Proc Natl Acad Sci USA.

[b96] Starkey Lewis PJ, Dear J, Platt V (2011). Circulating microRNAs as potential markers of human drug-induced liver injury. Hepatology.

[b97] Zhang Y, Jia Y, Zheng R (2010). Plasma microRNA-122 as a biomarker for viral-, alcohol-, and chemical-related hepatic diseases. Clin Chem.

[b98] Ladeiro Y, Couchy G, Balabaud C (2008). MicroRNA profiling in hepatocellular tumors is associated with clinical features and oncogene/tumor suppressor gene mutations. Hepatology.

[b99] Yeligar S, Tsukamoto H, Kalra VK (2009). Ethanol-induced expression of ET-1 and ET-BR in liver sinusoidal endothelial cells and human endothelial cells involves hypoxia-inducible factor-1alpha and microrNA-199. J Immunol.

[b100] Dolganiuc A, Petrasek J, Kodys K (2009). MicroRNA expression profile in Lieber-DeCarli diet-induced alcoholic and methionine choline deficient diet-induced nonalcoholic steatohepatitis models in mice. Alcohol Clin Exp Res.

[b101] Avissar M, McClean MD, Kelsey KT (2009). MicroRNA expression in head and neck cancer associates with alcohol consumption and survival. Carcinogenesis.

[b102] Dippold RP, Vadigepalli R, Gonye GE (2012). Chronic ethanol feeding enhances miR-21 induction during liver regeneration while inhibiting proliferation in rats. Am J Physiol Gastrointest Liver Physiol.

[b103] Dippold RP, Vadigepalli R, Gonye GE (2013). Chronic ethanol feeding alters miRNA expression dynamics during liver regeneration. Alcohol Clin Exp Res.

[b104] Liu J, Carmell MA, Rivas FV (2004). Argonaute2 is the catalytic engine of mammalian RNAi. Science.

[b105] Hutvagner G, Zamore PD (2002). A microRNA in a multiple-turnover RNAi enzyme complex. Science.

[b106] Filipowicz W, Bhattacharyya SN, Sonenberg N (2008). Mechanisms of post-transcriptional regulation by microRNAs: are the answers in sight?. Nat Rev Genet.

